# Nitroheterocyclic drugs cure experimental *Trypanosoma cruzi* infections more effectively in the chronic stage than in the acute stage

**DOI:** 10.1038/srep35351

**Published:** 2016-10-17

**Authors:** Amanda Fortes Francisco, Shiromani Jayawardhana, Michael D. Lewis, Karen L. White, David M. Shackleford, Gong Chen, Jessica Saunders, Maria Osuna-Cabello, Kevin D. Read, Susan A. Charman, Eric Chatelain, John M. Kelly

**Affiliations:** 1 Department of Pathogen Molecular Biology, London School of Hygiene and Tropical Medicine, Keppel Street, London WC1E 7HT, UK; 2 Drugs for Neglected Diseases Initiative (DNDi), 15 Chemin Louis-Dunant, 1202 Geneva, Switzerland; 3 Centre for Drug Candidate Optimisation, Monash University, 381 Royal Parade, Parkville 3052, Australia; 4 Drug Discovery Unit, Division of Biological Chemistry and Drug Discovery, School of Life Sciences, University of Dundee, Dundee DD1 5EH, UK

## Abstract

The insect-transmitted protozoan parasite *Trypanosoma cruzi* is the causative agent of Chagas disease, and infects 5–8 million people in Latin America. Chagas disease is characterised by an acute phase, which is partially resolved by the immune system, but then develops as a chronic life-long infection. There is a consensus that the front-line drugs benznidazole and nifurtimox are more effective against the acute stage in both clinical and experimental settings. However, confirmative studies have been restricted by difficulties in demonstrating sterile parasitological cure. Here, we describe a systematic study of nitroheterocyclic drug efficacy using highly sensitive bioluminescence imaging of murine infections. Unexpectedly, we find both drugs are more effective at curing chronic infections, judged by treatment duration and therapeutic dose. This was not associated with factors that differentially influence plasma drug concentrations in the two disease stages. We also observed that fexinidazole and fexinidazole sulfone are more effective than benznidazole and nifurtimox as curative treatments, particularly for acute stage infections, most likely as a result of the higher and more prolonged exposure of the sulfone derivative. If these findings are translatable to human patients, they will have important implications for treatment strategies.

Chagas disease is caused by the insect-transmitted protozoan *Trypanosoma cruzi* and is the most important parasitic infection in Latin America, affecting 5–8 million people^1^. It is also becoming a global problem, with increasing numbers of symptomatic cases in non-endemic areas, including the USA and Europe^2^,^3^. The initial acute stage of Chagas disease is usually relatively mild, although in children it can be severe, and sometimes fatal. With the development of a cellular immune response, parasitemia is suppressed, but sterile immunity is not achieved. Initially, the chronic infection phase is asymptomatic, but ~30% of patients eventually develop pathology, often decades later. Cardiomyopathy occurs in the majority of these individuals, whilst a minority suffer digestive tract megasyndromes^4^,^5^.

The nitroheterocyclic compounds benznidazole and nifurtimox are the front-line Chagas disease drugs^6^,^7^. Unfortunately, they display a range of toxic side-effects, which can impact negatively on patient compliance. Furthermore, both require bioactivation by the same parasite nitroreductase, a potential source of cross-resistance^8^,^9^. Treatment failures are common and new drugs are urgently required. Recent clinical trials have had disappointing outcomes. Posaconazole, a potent ergosterol biosynthesis inhibitor, was found to display limited curative potential against chronic infections^10^, and benznidazole, although partially^11^ or highly^10^ effective at achieving parasitological cure, showed no significant benefit in patients who had already developed advanced chagasic cardiomyopathy^11^.

There is general consensus that nitroheterocylic drugs are more effective against *T. cruzi* infections during the acute stage than in the chronic stage. Although widely quoted^12^,^13^,^14^,^15^,^16^,^17^,^18^,^19^,^20^,^21^, there have been few systematic studies to support this assertion. One of the major problems has been the difficulty in unequivocally demonstrating sterile cure, both in human patients and animal models. To increase the accuracy and reproducibility of drug testing, we developed highly sensitive bioluminescence methodology based on the expression by trypanosomes of a red-shifted luciferase reporter^22^,^23^,^24^. This *in vivo* imaging procedure has a limit of detection of 100–1000 parasites, and facilitates the real-time tracking of parasite burden in individual mice during long-term experimental infections. With the *T. cruzi* CL Brener-BALB/c mouse-parasite combination, the parasite burden peaks 14 days post-infection, and then resolves to the chronic phase over the next 30–40 days^23^. Infections persist for at least a year in dynamic equilibrium, at levels 2–3 orders of magnitude below the acute stage. Parasites are pan-tropic in the acute stage, but in chronically infected mice, the large intestine and stomach are the primary reservoir sites, a feature that also occurs for other parasite-mouse genotype combinations. Transient bioluminescent foci can also be detected at peripheral sites during chronic infections, which fluctuate in a spatiotemporally dynamic manner, appearing and disappearing over a period of hours. This bioluminescence imaging system is more reliable than PCR-based approaches for tracking experimental *T. cruzi* infections and for confirming parasitological cure^25^.

Here, we describe the use of this predictive model to undertake a detailed comparison of the efficacy of the nitroheterocyclic agents benznidazole, nifurtimox, fexinidazole and fexindazole sulfone against acute and chronic *T. cruzi* infections.

## Results

### Nitroheterocyclic drugs cure chronic stage infections more effectively than acute stage infections

**Figure 1:**
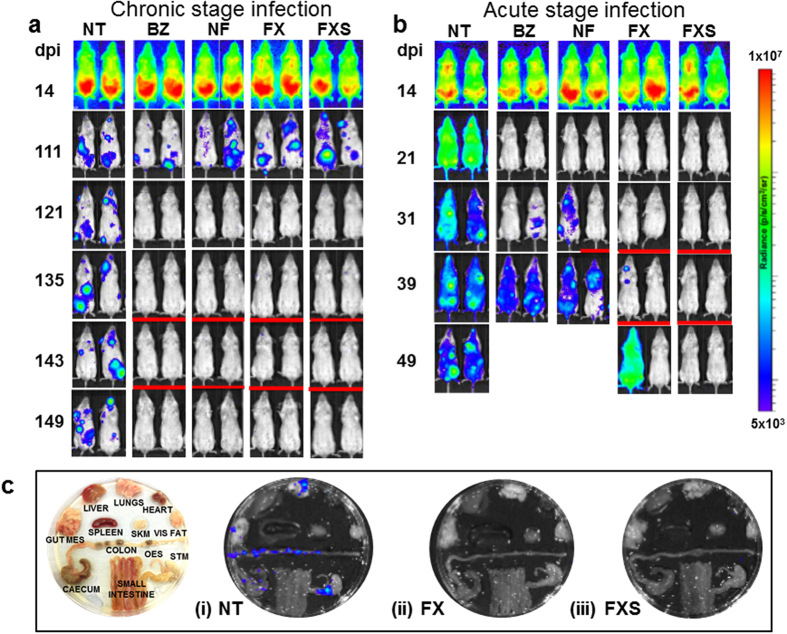
Treatment of chronic (**a**) and acute (**b**) stage *Trypanosoma cruzi* infections with nitroheterocyclic drugs assessed by *in vivo* imaging. BALB/c mice, infected with bioluminescent CL Brener parasites, were treated once daily by oral gavage for 5 days at 100 mg kg^−1^ with benznidazole (BZ), nifurtimox (NF), fexinidazole (FX), or fexinidazole sulfone (FXS). Treatment was initiated 114 days (chronic) or 14 days (acute) post-infection (see Table 1 for numbers of mice). Representative ventral images of 2 mice are shown for each drug, at various points post-infection. NT, non-treated (vehicle only). Mice with low/background levels of bioluminescence were immunosuppressed by cyclophosphamide (Methods), starting 135 or 31 days post-infection and are underlined with a red bar. All acute stage mice treated with BZ or NF were non-cured, and only immunosuppressed where necessary to confirm outcome. In acute stage infections, examples of both curative and non-curative outcomes with FX are shown. (**c**) Assessment of drug activity against acute stage infections by e*x vivo* imaging. Representative images of organs/tissues isolated 50 days post-infection (Methods) from NT (i), FX-treated (ii) and FXS-treated (iii) mice are shown. The locations of specific organs/tissues are illustrated in the left-hand image. The heat-map is on a log10 scale and indicates intensity of bioluminescence from low (blue) to high (red); the minimum and maximum radiances for the pseudocolour scale are shown.

Using the *T. cruzi* CL Brener-BALB/c model, we found that chronic infections could be cured with 5 daily oral doses of 100 mg kg^−1^ benznidazole (ref. 25, Table 1, Fig. 1a). Drug efficacy was assessed by both *in vivo* and *ex vivo* imaging, with cyclophosphamide-induced immunosuppression to enhance the reactivation of any residual infection (Methods). When we applied this same treatment regimen to mice at the peak of the acute stage, none of the 30 mice tested were cured (Table 1, Fig. 1b). Although treatment initiated 14 days post-infection resulted in a rapid drop in the bioluminescence-inferred parasite burden, this was transient, with a rapid return to levels similar to those in non-treated mice. Treatment of acute stage infections had to be extended to 20 days to achieve curative outcomes comparable to 5 days treatment in the chronic stage (Table 1).

Using the 5 day protocol, we also found nifurtimox to be highly effective at curing chronic infections, with 9/10 mice remaining bioluminescence negative after immunosuppression (Fig. 1a). In contrast, non-curative outcomes were observed in all cases when acute stage infections were treated with 100 mg kg^−1^ nifurtimox (Table 1, Fig. 1b). Therefore, with the 5 day treatment protocol, both of the front-line Chagas disease drugs are more effective against experimental infections in the chronic stage.

We next tested fexnidazole and its metabolite fexinidazole sulfone. Previous studies suggested that these nitroimidazoles might have greater *in vivo* activity than benznidazole^26^,^27^. When chronically infected mice were treated for 5 days with oral doses of 100 mg kg^−1^, there was a 100% cure rate in each case (Table 1, Fig. 1a). A similar outcome was observed when acute stage infections were treated with fexinidazole sulfone (Fig. 1b,c). Fexinidazole also showed significant efficacy during acute stage infections, curing 4/6 mice.

**Figure 2:**
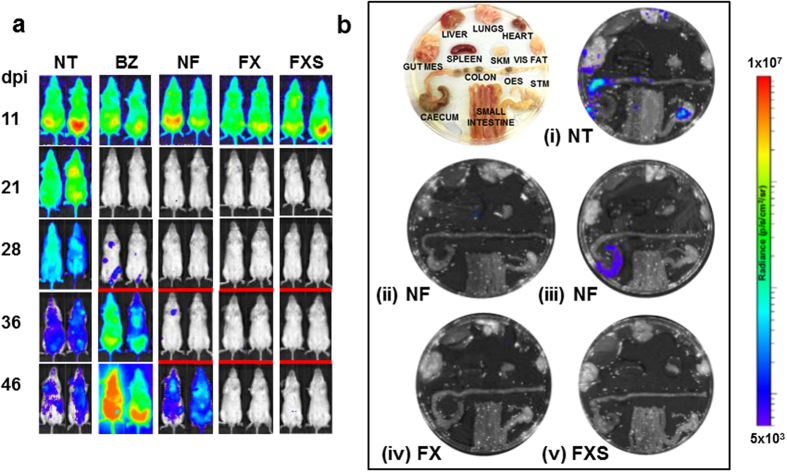
Effectiveness of nitroheterocyclic drugs in treating acute stage *Trypanosoma cruzi* infections when administered for 5 days at 50 mg kg^−1^ bid. (**a**) *In vivo* imaging of BALB/c mice infected with bioluminescent *T. cruzi* (CL Brener strain) and treated twice daily by the oral route with benznidazole (BZ), nifurtimox (NF), fexinidazole (FX), or fexinidazole sulfone (FXS). Treatment was initiated 14 days post-infection (n = 6). Representative ventral images of 2 mice are shown for each drug, at various time points. NT, non-treated (vehicle only). Where necessary, to confirm curative outcomes, mice were immunosuppressed by cyclophosphamide treatment on days 28, 32 and 36 post-infection (underlined with a red bar). (**b**) Assessment of drug activity by *ex vivo* imaging. Representative images of organs/images isolated 50 days post-infection from NT (i), NF-treated (ii and iii), FX-treated (iv), and FXS-treated (v) mice. In the case of NF treatment, examples of cured (ii) and non-cured (iii) mice are illustrated.

To further explore the 5 day treatment protocol for acute stage infections, we administered nitroheterocyclic drugs as two daily doses of 50 mg kg^−1^. With this schedule, benznidazole again failed to cure any mice (0/6) (Table 1, Fig. 2). Nifurtimox was more effective at reducing parasite burden, but only 1/6 mice was ultimately cured. In contrast, fexinidazole treatment was 100% successful (6/6) and fexinidazole sulfone curative in 5/6 cases. Fexinidazole sulfone was then investigated by administering single doses of 50 mg kg^−1^ for 5 days. With chronic stage infections, all 6 mice were cured. In contrast, none were cured when acute stage infections were treated, confirming the differential effectiveness of nitroheterocyclic drugs against the two disease stages (Table 1).

**Figure 3:**
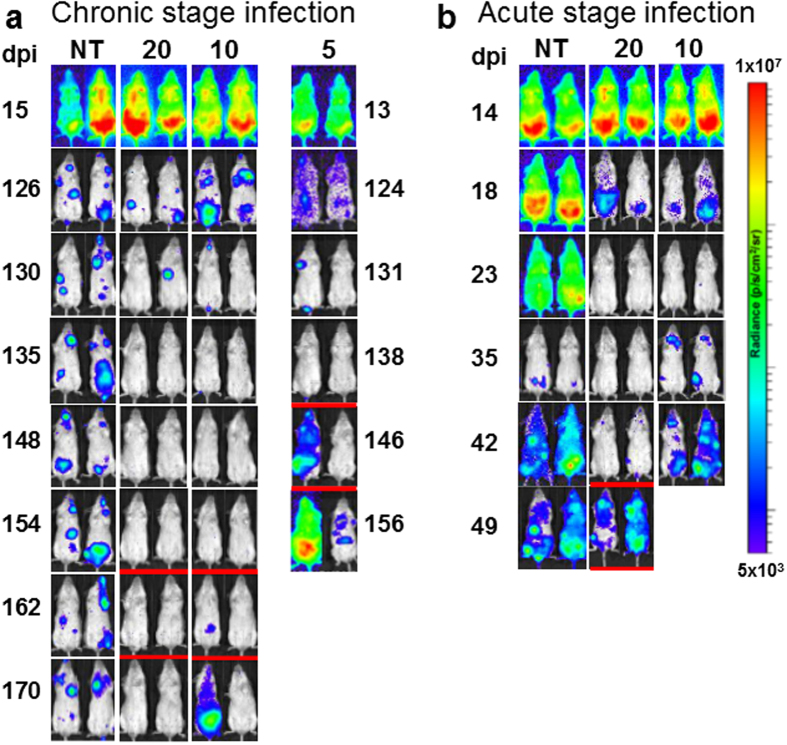
Treatment of chronic (**a**) and acute (**b**) stage *Trypanosoma cruzi* infections with 30 mg kg^−1^ benznidazole. Treatment of infected BALB/c mice (n = 6) with bioluminescent parasites (CL Brener strain) was initiated 124 or 126 days (chronic), or 14 days (acute) post-infection, once daily by the oral route, for the number of days indicated. Representative ventral images of the same pair of mice are shown for each drug, at various points post-infection. NT, non-treated (vehicle only). Where required to confirm curative outcomes, mice were immunosuppressed by cyclophosphamide treatment on days 42 and 46 (acute stage experiment), 138, 142 and 146 (chronic stage experiment, 5 days treatment), and 154, 158 and 162 (chronic stage experiment, 10 and 20 days treatment) (underlined with red bar). All mice scored as cured were bioluminescence negative by both *in vivo* and *ex vivo* imaging.

When chronically infected mice were treated daily with 30 mg kg^−1^ benznidazole for 20 days, there was a 100% cure rate (6/6) (Table 1, Fig. 3a). Against acute stage infections however, the 30 mg kg^−1^ dosing regimen was less effective, with only 2/6 mice cured (Fig. 3b). This stage-specific trend was also observed when treatment duration was reduced to 10 days; 4/6 chronically infected mice were cured, compared to 100% treatment failure (0/6) in the acute stage (Table 1, Fig. 3a,b). When 5 days treatment was assessed, we found that benznidazole could reduce bioluminescence to background levels in most chronically infected mice, but the effect was transient and the parasite burden rebounded, particularly after immunosuppression. In this model therefore, daily dosing with 30 mg kg^−1^ benznidazole is more effective against chronic than acute stage infections, with the duration of treatment being a major factor in determining outcome. Treatment of chronic infections for 20 days with doses as low as 10 mg kg^−1^ also resulted in a significant reduction in parasite burden, although in this case, only 1 out of 6 mice was ultimately cured (Table 1). Nifurtimox, fexinidazole and fexinidazole sulfone displayed significant activity at 30 mg kg^−1^ against chronic stage infections. For example, 10 days treatment of chronic infections with 30 mg kg^−1^ nifurtimox produced a higher cure rate (5/6) than 10 days treatment of acute stage infections with 100 mg kg^−1^ (1/6). As with benznidazole, restricting the treatment period to 5 days, reduced drug efficacy in all cases (Table 1).

### Greater efficacy against chronic stage infections is not a model-specific phenomenon

**Figure 4:**
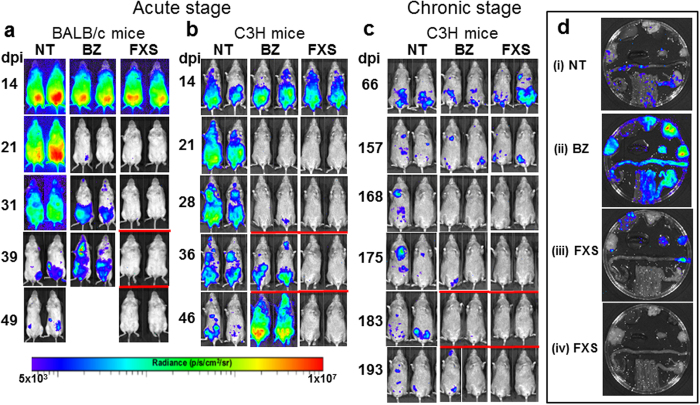
Nitroheterocylic drug activity in other experimental models of Chagas disease. (**a**) BALB/c mice infected with bioluminescent *T. cruzi* JR strain were treated with benznidazole (BZ) or fexinidazole sulfone (FXS) 14 days post-infection. NT, non-treated (vehicle only). Drugs were administered orally, for 5 days at 100 mg kg^−1^. FXS-treated mice were also immunosuppressed by cyclophosphamide treatment on days 32, 36 and 40 to promote the reactivation of any residual parasites (underlined with red bar). BZ was non-curative in all cases, (n = 6), whereas 4/5 mice treated with FXS were judged parasite-free after both *in vivo* and *ex vivo* imaging. (**b**) C3H mice infected with bioluminescent JR strain were treated orally with BZ or FXS 14 days post-infection, for 5 days at 100 mg kg^−1^. Where necessary, to confirm curative outcomes, mice were immunosuppressed by cyclophosphamide treatment on days 28, 32 and 36 post-infection (underlined with a red bar). BZ was non-curative (n = 5), whereas 4/5 mice treated with FXS were parasite-free after both *in vivo* and *ex vivo* imaging. (**c**) C3H mice, chronically infected with bioluminescent JR strain were treated orally with BZ or FXS for 5 days at 100 mg kg^−1^, commencing 161 days post-infection. Where required, cyclophosphamide treatment commencing on day 175, was used to promote reactivation. BZ-treatment cured 3/5 mice, whereas 5/5 mice were cured with FXS. (**d**) Assessment of drug-activity by *ex vivo* imaging. Organs/tissues from mice treated in the acute stage were removed 50 days post-infection (Methods) from NT (i), BZ-treated (ii) and FXS-treated (iii and iv) mice. Organs/tissues are arranged as described in Figs 1a and 2b. In the case of FXS treatment, examples of non-cured (iii) and cured (iv) mice are illustrated.

We investigated if our observations were relevant to other experimental models of Chagas disease. First, we assessed whether fexinidazole sulfone was also more effective than benznidazole in BALB/c mice infected with the JR strain of *T. cruzi*. The CL Brener (lineage TcVI) and JR (lineage TcI) strains are highly divergent^28^, although both strains display similar infection profiles in a BALB/c background when monitored by bioluminescence imaging^29^. Treatment of acute stage infections with 100 mg kg^−1^ benznidazole for 5 days resulted in a rapid knockdown in bioluminescence (Fig. 4a, Table 1). However, as with CL Brener infections, this effect was transient and non-curative (Figs 1b and 4a). When JR-infected BALB/c mice were treated with fexinidazole sulfone using the same dosing regimen, all but one of the mice were successfully cured (Fig. 4a). Therefore, at similar doses, fexinidazole sulfone is more effective than benznidazole as a treatment for acute stage infections in two diverse *T. cruzi* strains.

We next examined the efficacy of both drugs in C3H mice. In this strain, the parasite burden is typically higher and more disseminated than in BALB/c mice^29^. When acute stage JR-infections were assessed following 5 days treatment (100 mg kg^−1^), the outcomes were similar to those in the BALB/c strain, with 100% treatment failure in the case of benznidazole (n = 5), but curative outcomes in all but one of the mice treated with fexinidazole sulfone (Fig. 4, Table 2). In C3H mice, infections with the JR strain display an extended acute phase, reaching chronic stage equilibrium later than in BALB/c mice^29^. For assessment of efficacy against chronic infections, treatment (5 days, 100 mg kg^−1^) was therefore delayed until day 161. In these circumstances, fexinidazole sulfone cured 5/5 mice, with 3/5 cured in the case of benznidazole (Fig. 4c). Thus, *T. cruzi* infections are easier to cure in the chronic stage in two distinct murine/parasite models.

### Pharmacokinetics and drug efficacy

To relate treatment outcomes to drug exposure, we assessed plasma concentrations of nitroheterocyclic drugs following single oral doses. For all compounds, the measured blood to plasma ratio was ~1, the fraction unbound in the *in vitro* assay media was >0.9, and the fraction unbound in plasma was 0.7 for benznidazole, 0.5 for nifurtimox, and 0.4 for fexinidazole and fexinidazole sulfone. Given the low and comparable extent of binding, data comparisons were made using the total plasma concentration throughout.

**Figure 5:**
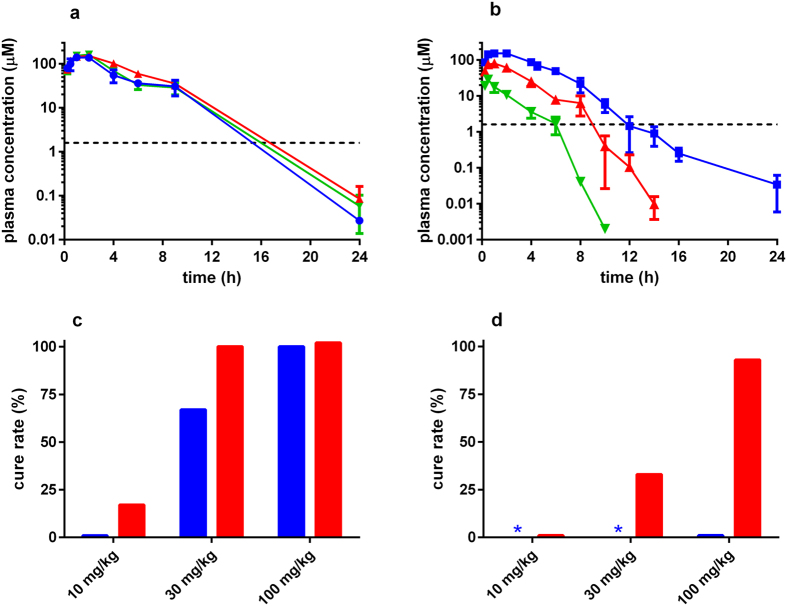
Pharmacokinetic and cure rate data for benznidazole. (**a**) Plasma concentration versus time profiles of benznidazole following a single 100 mg kg^−1^ dose in uninfected (blue), acute (red) and chronic (green) stage infected BALB/c mice. The *in vitro* IC_50_ is shown by the dashed line. (**b**) Plasma concentration versus time profiles following single doses of 10 (green), 30 (red), and 100 (blue) mg kg^−1^ benznidazole in uninfected BALB/c mice. (**c**) Cure rates for benznidazole following 10 days (blue bar) and 20 days (red bar) treatment in chronic stage infected mice. (**d**) Cure rates for benznidazole following 10 days (blue bar) and 20 days (red bar) treatment in acute stage infected mice. Because 100 mg kg^−1^ was ineffective with 10 days treatment in the acute stage, 30 and 10 mg kg^−1^ treatments were not tested (blue asterisks).

Although there are no reports of differences in pharmacokinetics between acute and chronic infections, we investigated if the disparity in efficacy between the two stages could be linked to drug exposure. Benznidazole concentrations in mouse plasma were measured in uninfected mice, and during acute and chronic infections, after a single oral dose of 100 mg kg^−1^. Plasma concentrations were comparable under all conditions (Fig. 5a), suggesting that the reduced ability to cure acute stage infections was not attributable to factors that influence drug exposure (Fig. 5b).

Plasma concentrations of benznidazole reached a C_max_ within 1 hour (Fig. 5b, Table 2), and then declined in a dose-dependent manner, dropping below the *in vitro* IC_50_ values after 6–12 hours (see also^26^). There was a clear association between benznidazole dose/exposure and effectiveness against both acute and chronic stage infections (Fig. 5c,d), although as noted above, the acute stage infections required a longer treatment duration.

**Figure 6:**
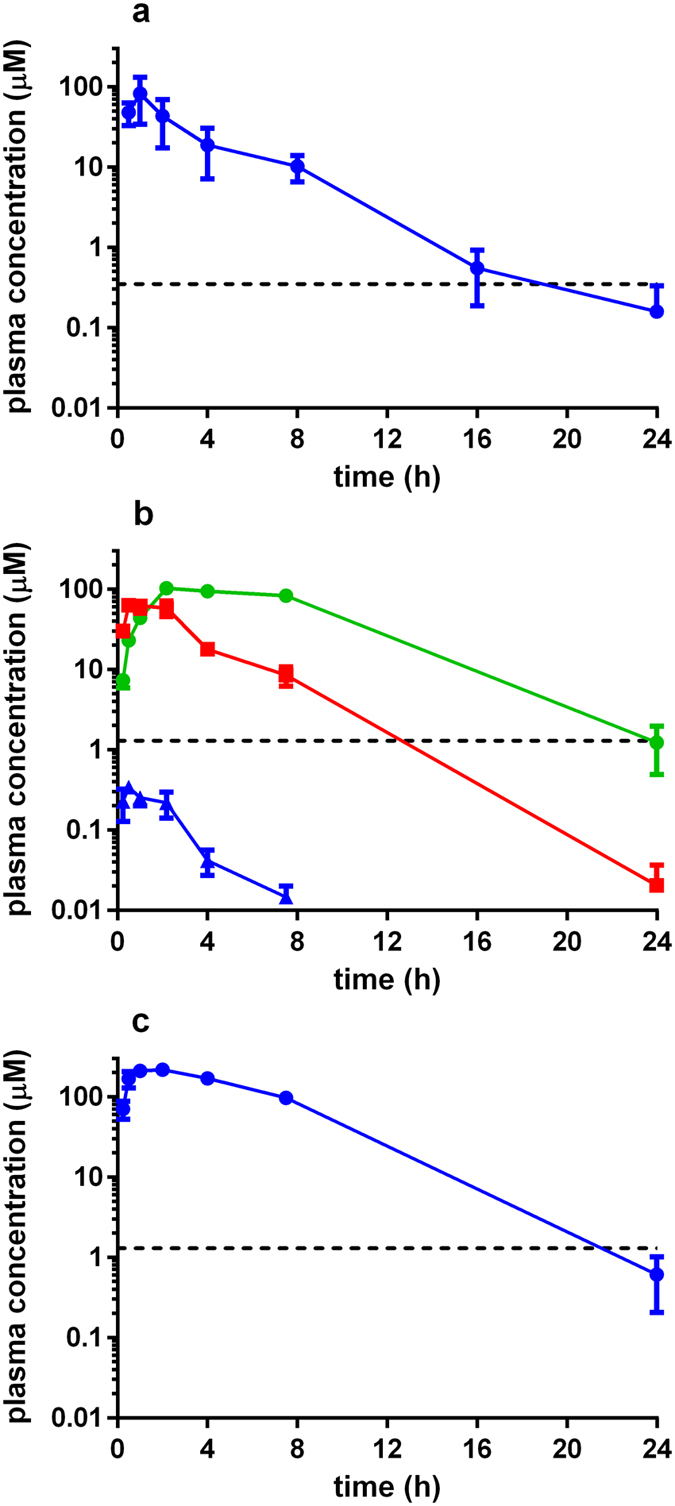
Pharmacokinetic data for (**a**) nifurtimox, (**b**) fexinidazole, and (**c**) fexinidazole sulfone following a single 100 mg kg^−1^ dose (Methods). For fexinidazole (**b**), profiles for the metabolites fexinidazole sulfone (green) and fexinidazole sulfoxide (red) are shown together with the parent compound (blue). Data for fexinidazole sulfone reproduced from ref. 27. The *in vitro* IC_50_ values are shown by the dashed lines.

When we investigated the PK properties of nifurtimox, we found that the C_max_ and AUC were lower than for benznidazole, but plasma concentrations remained above the *in vitro* IC_50_ for longer (Fig. 6a). This may contribute to the greater effectiveness of nifurtimox in reducing parasite burden when acute infections are treated with 50 mg kg^−1^ twice daily (Fig. 2), even though curative outcomes were limited. When fexinidazole plasma concentrations were assessed, the parent drug was detected at low concentrations (<1 μM), and only for a short period (Fig. 6b), however the two active metabolites, fexinidazole sulfoxide and sulfone^30^ were rapidly formed, with C_max_ values of 1–2 hours and 4–5 hours, respectively. Both metabolites remained above their *in vitro* IC_50_ for prolonged periods (12–14 hours for the sulfoxide, 24 hours for the sulfone). In mice where fexinidazole sulfone was administered directly, plasma concentrations reached a higher C_max_ than achieved in fexinidazole-treated mice (217 vs 103 μM), with a corresponding increase in the AUC (Table 2 and ref. 27). The prolonged exposure of the sulfone could in part explain why fexinidazole and fexinidazole sulfone have greater efficacy than benznidazole at the same dose (Table 1). The steep drop-off in efficacy against acute infections when the daily dose of fexinidazole sulfone was reduced from 100 to 50 mg kg^−1^, or when one daily dose at 50 mg kg^−1^ was administered instead of two, highlights the narrow window of drug exposure that determines treatment outcome in the 5 day regimen.

## Discussion

Drug tests against chronic Chagas disease (both experimental and clinical) have been problematic, mainly because it is difficult to unambiguously confirm parasitological cure, even with PCR-based methodologies^25^. The dynamic, focal nature of the infection, and the extremely low parasite burden have been confounding factors^23^,^29^. Here, we used highly sensitive bioluminescence imaging coupled with cyclophosphamide-mediated immunosuppression to provide a reliable and flexible approach for systematic and comparative studies of drug efficacy. Our major finding is that nitroheterocyclic drugs cure *T. cruzi* infections in murine models more readily in the chronic stage than in the acute stage. This result is contrary to the widely held view that chronic infections in humans are intrinsically more difficult to treat^12^,^13^,^14^,^15^,^16^,^17^,^18^,^19^,^20^,^21^. We were able to exclude factors that influence plasma concentration for this differential drug efficacy, at least in the case of benznidazole (Fig. 5a). An alternative explanation could be that the pan-tropic nature of acute stage infections^23^ requires drugs to access every organ and tissue at levels sufficient to produce a sterile cure. In chronic stage infections this might be easier to achieve, as the parasite burden is considerably lower and restricted to far fewer locations than in the acute phase. In mouse models, the colon and/or stomach are the major sites of parasite persistence, with other organs and tissues sporadically infected^29^.

Most *in vivo* drug testing has focused on acute stage infections, partly because it is more straightforward to monitor parasite burden^31^,^32^,^33^,^34^. From a clinical perspective however, the ability to cure chronic infections is the primary requirement^35^. Therefore, the differential stage-specific responses to nitroheterocyclic drugs highlight that chronic infections should be the major focus of studies in predictive animal models. In line with this, there is a need to systematically assess the extent to which drug-induced cure of chronic infections alleviates or prevents the development of cardiac pathology. There is strong evidence that successful treatment of acute stage infections is associated with improved prognosis^36^,^37^,^38^,^39^. However, with chronic infections, the evidence is less certain^40^. For example, in the BENEFIT trial, improvements in clinical outcomes related to cardiac cardiomyopathy were not observed 5 years after benznidazole therapy^11^, although aspects of this study are the subject of debate^41^,^42^,^43^,^44^. The availability of improved predictive models of chronic Chagas disease now provides an experimental platform to dissect the link between curative therapy and disease pathology. Data from such studies will be invaluable for informing and guiding the design of future clinical trials.

The current front-line drugs are used against acute stage infections, with cure rates of 80–90%^19^. Because of the complex and long-term nature of Chagas disease, and difficulties in establishing parasitological cure, there have been few rigorous studies to optimise treatment of chronic infections^40^. Therapeutic schedules have been based on regimens that proved relatively successful against the acute stage. However, a combination of severe side-effects and treatment length (60–90 days), in a context where the infection is often asymptomatic, can lead to compliance issues that impact on curative outcome. The data from this study, which demonstrate that chronic infections in mouse models are more easily cured by nitroheterocyclic drugs than acute stage infections (Table 1), suggest the possibility of reducing treatment duration and drug dose.

Although fexinidazole has been advanced into Phase II clinical trials against *T. cruzi* infection, the study was not completed because of tolerability issues^45^. Here, we demonstrate that fexinidazole and fexinidazole sulfone are more efficacious than both of the front-line drugs in murine models at equivalent doses, particularly in the acute stage. For example, 5 days treatment with 50 mg kg^−1^ bid, is almost always curative with fexinidazole and fexinidazole sulfone, but rarely so with benznidazole or nifurtimox (Table 1). Similarly, the effectiveness of the fexinidazole compounds is evident when administered in single daily doses of 100 mg kg^−1^. The PK studies suggest that this enhanced activity could be linked to higher and more prolonged plasma exposure than is achievable with either benznidazole or nifurtimox (Figs 5 and 6; Table 2), thereby allowing more effective targeting of parasites in a wider range of organs and tissues. However, these PK-based inferences are complicated by the fact that the trypanocidal properties of nitroheterocyclic drugs result from reactive metabolites generated within the parasite following nitroreductase-mediated activity^8^,^46^,^47^. We also cannot exclude the possibility of exposure changes with repeat administration, given that the PK studies were all single dose experiments. Whatever the determinant of efficacy, our results highlight the potential for reducing the treatment duration and/or therapeutic dose of fexinidazole and fexinidazole sulfone as a means of circumventing toxicity and compliance issues.

In summary, the application of highly sensitive imaging technology to predictive models of Chagas disease provides new insights into drug efficacy. Of particular importance is the finding that *T. cruzi* infections are more readily cured in the chronic stage. It will be important to establish if these observations are transferrable to a clinical setting.

## Methods

### Mice

Infection experiments were approved by the LSHTM Ethics Committee and performed under UK Home Office licence PPL70/8207. All methods and manipulations were performed in accordance with the requirements of this licence. Female BALB/c and C3H/HeN mice were obtained from Charles River (UK) and CB17 SCID mice were bred in-house. Mice were maintained as described previously^23^ and were aged 8–12 weeks when infected with bioluminescent CL Brener^23^ or JRcl4 strains^29^. Typically, 1 × 10^4^ bloodstream trypomastigotes (BTs) in 0.2 ml PBS were used to infect SCID mice via intraperitoneal (i.p.) inoculation. Parasitaemic blood from these mice was obtained 2–3 weeks later and adjusted to 5 × 10^3^ BTs ml^−1^ with PBS. Mice were then infected i.p. with 1 × 10^3^ BTs^23^. On occasions (~10%), infection with the JR strain led to a fatal outcome, prior to the initiation of treatment. These mice were not included in the data set (Table 1).

### Parasites

*T. cruzi* strains CL Brener and JRcl4 were grown as epimastigotes at 28 °C^26^. Metacyclic trypomastigotes were obtained by transfer to Graces-IH medium^48^ and harvested after 4–7 days. Tissue culture trypomastigotes were obtained from infected L6 rat myoblasts^29^. Bioluminescent parasites expressing the firefly luciferase *PpyRE9h* gene^22^ were generated as described^23^,^24^.

### Drug Treatment

Benznidazole, nifurtimox, fexinidazole and fexinidazole sulfone were synthesized by Epichem Pty Ltd., Australia, and prepared at 5 mg ml^−1^ in an aqueous suspension vehicle containing 5% (v/v) DMSO, 0.5% (w/v) hydroxypropyl methylcellulose, 0.5% (v/v) benzyl alcohol and 0.4% (v/v) Tween 80. Drugs were administered by oral gavage (~200 μl), and vehicle only was administered to control mice. To detect residual infection, mice were immunosuppressed with cyclophosphamide (200 mg kg^−1^) by i.p. injection every 4 days for a maximum of 3 doses.

### *In vivo* bioluminescence imaging

Mice were injected i.p. with 150 mg kg^−1^ d-luciferin in Dulbecco’s Ca^2+^/Mg^2+^ free PBS, then anaesthetized using 2.5% (v/v) isofluorane in oxygen. Images were obtained using an IVIS Lumina II system (Caliper Life Science) 10–20 minutes after d-luciferin administration. Exposure times varied from 30 seconds to 5 minutes, depending on signal intensity. To estimate parasite burden, whole body regions of interest were drawn using LivingImage v4.3 to quantify bioluminescence expressed as total flux (photons/second; p/s). The detection threshold was established from uninfected mice^23^. Animals with bioluminescence intensity below 5 × 10^3 ^p/s/sr/cm^2^ in both dorsal and ventral images following immunosuppression were designated cured, subject to confirmation by *ex vivo* assessment.

### Assessing treatment efficacy by *ex vivo* imaging

Organs/tissue samples were assessed by *ex vivo* imaging^23^,^25^. Briefly, mice were injected i.p. with 150 mg kg^−1^ d-luciferin, then sacrificed by ex-sanguination under terminal anaesthesia 7 minutes later. They were perfused with 10 ml 0.3 mg ml^−1^ d-luciferin in PBS via the heart. Organs/tissues were excised, transferred to a Petri dish, soaked in 0.3 mg ml^−1^ d-luciferin, then imaged. Routinely, the carcass was assessed for bioluminescence associated with skin, skeletal muscle or remaining adipose tissue.

### Pharmacokinetic analysis

Pharmacokinetic (PK) studies in non-infected mice conformed to the Australian Code of Practice for the Care and Use of Animals for Scientific Purposes and were approved by the Monash Institute of Pharmaceutical Sciences Animal Ethics Committee. PK studies were conducted using infected and non-infected female BALB/c (benznidazole) or non-infected female (benznidazole, fexinidazole and fexinidazole sulfone) and male (nifurtimox) Swiss outbred mice. Drugs were administered by oral gavage. For the efficacy studies, blood samples (10 μl) were collected from the tail vein into tubes containing MilliQ water (20 μl) at 0.25, 0.5, 1, 2, 4, 6, 9, and 24 hours post-dose. Samples were subjected to 3x freeze/thaw cycles in liquid nitrogen before bioanalysis. The level of benznidazole in mouse blood was determined using UPLC-MS/MS (Waters Xevo TQs) following protein precipitation with acetonitrile. Blood concentrations were converted to plasma concentrations using the measured blood to plasma ratio of ~1.

For the parallel PK studies, a maximum of two blood samples were collected from each mouse (with three mice per time-point) by submandibular bleed and terminal cardiac puncture into heparinised tubes. For benznidazole, nifurtimox and fexinidazole, samples were collected for up to 24 hours post-dose, whereas for fexinidazole sulfone, samples were collected over 48 hours. Plasma was separated by centrifugation and stored at −80 °C (maximum two weeks). For analysis, samples were thawed, proteins precipitated, and the supernatant analysed by LC/MS with quantitation by comparison to a calibration curve prepared in blank plasma. Blood:plasma partitioning ratios in mouse plasma, and the fraction unbound in plasma and *in vitro* assay media binding, were assessed as described^29^.

For both efficacy and PK studies, plasma concentration versus time data were analysed by non-compartmental PK methods to obtain the maximum concentration (C_max_), the time to reach C_max_ (T_max_), and the area under the plasma concentration time curve to infinity (AUC_∞_).

## Additional Information

**How to cite this article**: Francisco, A. F. *et al*. Nitroheterocyclic drugs cure experimental *Trypanosoma cruzi* infections more effectively in the chronic stage than in the acute stage. *Sci. Rep.* **6**, 35351; doi: 10.1038/srep35351 (2016).

## Figures and Tables

**Table 1:** Nitroheterocyclic drug efficacy in BALB/c and C3H mice infected with *Trypanosoma cruzi* CL Brener and JR strains.

			Chronic Infection Cure Rate			Acute Infection Cure Rate		
			*T. cruzi* CL Brener strain			Treatment length			Treatment length		
Drug	Mouse strain	Dose (mg kg^−1^)				5 days	10 days	20 days	5 days	10 days	20 days
**BZ**	BALB/c	10 qd	—	0% (0/6)	17% (1/6)	—	0% (0/6)	0% (0/6)
	BALB/c	30 qd	0% (0/6)	67% (4/6)	100% (6/6)	—	0% (0/6)	33% (2/6)
	BALB/c	100 qd	100% (11/11)*	100%(15/15)*	100%(11/11)*	0% (0/30)	0% (0/6)	93%(14/15)
	BALB/c	50 bid	—	100% (6/6)	—	0% (0/6)	—	—
**NF**	BALB/c	30 qd	33% (2/6)	83% (5/6)	—	—	—	—
	BALB/c	100 qd	90% (9/10)	—	—	0% (0/6)	17 (1/6)	—
	BALB/c	50 bid	—	—	—	17% (1/6)	—	—
**FX**	BALB/c	30 qd	50% (3/6)	100% (6/6)	—	—	—	—
	BALB/c	100 qd	100% (8/8)	—	—	67% (4/6)	100 (6/6)	—
	BALB/c	50 bid	—	—	—	100% (6/6)	—	—
**FXS**	BALB/c	30 qd	17% (1/6)	100% (6/6)	—	0% (0/6)	—	—
	BALB/c	50 qd	100% (6/6)	—	—	0% (0/6)	—	—
	BALB/c	100 qd	100% (7/7)	—	—	100%(15/15)	—	—
	BALB/c	50 bid	—	—	—	83% (5/6)	—	—
***T. cruzi*** **JR strain**								
**BZ**	BALB/c	100 qd	—	—	—	0% (0/6)	—	—
	C3H	100 qd	60% (3/5)	—	—	0% (0/5)	—	—
**FXS**	BALB/c	100 qd	—	—	—	80% (4/5)	—	—
	C3H	100 qd	100% (5/5)	—	—	80% (4/5)	—	—

*includes data reported previously in ref. 25 qd; *quaque die (once daily), bid; bis in die* (twice daily).

Following treatment, mice in the acute or chronic stage were monitored by bioluminescence imaging (Methods, Figs 1, 2, 3 and 4). Mice were only designated as cured if they were bioluminescence negative by both *in vivo* and *ex vivo* imaging, following immunosuppressive treatment. BZ = benznidazole; NF = nifurtimox; FX = fexinidazole; FXS = fexinidazole sulfone.

**Table 2:** Pharmacokinetic parameters following single dose administration to uninfected BALB/c (benznidazole) or Swiss outbred (nifurtimox, fexinidazole, fexinidazole sulfone) mice.

Drug	C_max_ (μM)	T_max_ (h)	AUC∞ (μM.h)
**Benznidazole**			
10 mg kg^−1^	27.0	0.5	51.1
30 mg kg^−1^	80.4	1.0	252
100 mg kg^−1^	153	1.0	726
**Nifurtimox**			
100 mg kg^−1^	52.5	1.0	179
**Fexinidazole**			
100 mg kg^−1^	0.30 (FX)	0.5 (FX)	0.94 (FX)
	63.3 (FXSX)	0.5 (FXSX)	304 (FXSX)
	103 (FXS)	2.2 (FXS)	1300 (FXS)
**Fexinidazole sulfone**			
100 mg kg^−1^	217	2.0	2010

Data for fexinidazole sulfone were taken from ref. 27. FX = fexinidazole; FXSX = fexinidazole sulfoxide; FXS = fexinidazole sulfone. Note that data for nifurtimox were generated in male mice and gender-related differences in PK properties cannot be ruled out.

## References

[b1] Kirchhoff L. V. Epidemiology of American trypanosomiasis (Chagas disease). Adv. Parasitol. 75, 1–15 (2011). 21820549 10.1016/B978-0-12-385863-4.00001-0

[b2] Bern C. & Montgomery S. P. An estimate of the burden of Chagas disease in the UnitedStates. Clin. Infect. Dis. 49, e52–e54 (2009). 19640226 10.1086/605091

[b3] www.who.int/neglected_diseases/integrated_media_chagas_statement/en/index.

[b4] Carod-Artal F. J. Trypanosomiasis, cardiomyopathy and the risk of ischemic stroke. Expert Rev. Cardiovasc. Ther. 8, 717–728 (2010). 20450304 10.1586/erc.10.33

[b5] Jabari S., de Oliveira E. C., Brehmer A. & da Silveira A. B. Chagasic megacolon: entericneurons and related structures. Histochem. Cell. Biol. 42, 235–244 (2014). 10.1007/s00418-014-1250-x PMC4133073 25059649

[b6] Salomon C. J. First century of Chagas’ disease: an overview on novel approaches to nifurtimox and benzonidazole delivery systems. J. Pharm. Sci. 101, 888–894 (2012). 22161779 10.1002/jps.23010

[b7] Gaspar L. . Current and future chemotherapy for Chagas disease. Curr. Med. Chem. 22, 4293–4312 (2015). 26477622 10.2174/0929867322666151015120804

[b8] Wilkinson S. R., Taylor M. C., Horn D., Kelly J. M. & Cheeseman I. A mechanism for cross-resistance to nifurtimox and benznidazole in trypanosomes. Proc. Natl. Acad. Sci. USA 105, 5022–5027 (2008). 18367671 10.1073/pnas.0711014105 PMC2278226

[b9] Mejia A. M. . Benznidazole-resistance in *Trypanosoma cruzi* is a readily acquired trait that can arise independently in a single population. J. Inf. Dis. 206, 220–228 (2012). 22551809 10.1093/infdis/jis331 PMC3379838

[b10] Molina I. . Randomized trial of posaconazole and benznidazole for chronic Chagas disease. N. Engl. J. Med. 370, 1899–1908 (2014). 24827034 10.1056/NEJMoa1313122

[b11] Morillo C. A. . Randomized trial of benznidazole for chronic Chagas’ cardiomyopathy. N. Engl. J. Med. 373, 1295–1306 (2015). 26323937 10.1056/NEJMoa1507574

[b12] Rodriques Coura J. & de Castro S. L. A critical review on Chagas disease chemotherapy. Mem. Inst. Oswaldo Cruz 97, 3–24 (2002). 11992141 10.1590/s0074-02762002000100001

[b13] Croft S. L., Barrett M. P. & Urbina J. A. Chemotherapy of trypanosomiases and leishmaniasis. Trends Parasitol. 21, 508–512 (2005). 16150644 10.1016/j.pt.2005.08.026

[b14] Wilkinson S. R. & Kelly J. M. Trypanocidal drugs: mechanisms, resistance and new targets. Expert Rev. Molec. Med. 11, e31 pp1–24 (2009). 19863838 10.1017/S1462399409001252

[b15] Urbina J. A. Specific chemotherapy of Chagas disease: Relevance, current limitations and new approaches. Acta Trop. 115, 55–68 (2010). 19900395 10.1016/j.actatropica.2009.10.023

[b16] Rassi A. Jr., Rassi A. & Marin-Neto J. A. Chagas disease. Lancet Infect. Dis. 10, 556–570 (2010). 20670903 10.1016/S1473-3099(10)70098-0

[b17] Jackson Y. . Tolerance and safety of nifurtimox in patients with chronic chagas disease. Clin. Infect. Dis. 51, e69–e75 (2010). 20932171 10.1086/656917

[b18] Wilkinson S. R., Bot C., Kelly J. M. & Hall B. S. Trypanocidal activity of nitroaromatic prodrugs: current treatments and future perspectives. Curr. Topics Med. Chem. 11, 2072–2084 (2011). 10.2174/156802611796575894 21619510

[b19] Guedes P. M., Silva G. K., Gutierrez F. R. & Silva J. S. Current status of Chagas disease chemotherapy. Expert Rev. Anti-infect. Ther. 9, 609–620 (2011). 21609270 10.1586/eri.11.31

[b20] Bern C. Antitrypanosomal therapy for chronic Chagas’ disease. N. Engl. J. Med. 364, 2527–2534 (2011) 21714649 10.1056/NEJMct1014204

[b21] Bermudez J., Davies C., Simonazzi A., Pablo Real J. & Palma S. Current drug therapy and pharmaceutical challenges for Chagas disease. Acta Trop. 30, 1–16 (2015). 10.1016/j.actatropica.2015.12.017 26747009

[b22] Branchini B. R. . Red-emitting luciferases for bioluminescence reporter and imaging applications. Anal. Biochem. 396, 290–297 (2010). 19748472 10.1016/j.ab.2009.09.009

[b23] Lewis M. D. . Bioluminescence imaging of chronic *Trypanosoma cruzi* infections reveals tissue-specific parasite dynamics and heart disease in the absence of locally persistent infection. Cell. Microbiol. 16, 1285–1300 (2014). 24712539 10.1111/cmi.12297 PMC4190689

[b24] Lewis M. D., Fortes Francisco A., Taylor M. C. & Kelly J. M. A new experimental model for assessing drug efficacy against *Trypanosoma cruzi* infection based on highly sensitive *in vivo* imaging. J. Biomolec. Screening 20, 36–43 (2015). 10.1177/1087057114552623 PMC4361455 25296657

[b25] Fortes Francisco A. . The limited ability of posaconazole to cure both acute and chronic *Trypanosoma cruzi* infections revealed by highly sensitive *in vivo* imaging. Antimicrob. Agents Chemother. 59, 4653–4661 (2015). 26014936 10.1128/AAC.00520-15 PMC4505219

[b26] Bahia M. T. . Fexinidazole: a potential new drug candidate for Chagas disease. PLoS Negl. Trop. Dis. 6, e1870 (2012). 23133682 10.1371/journal.pntd.0001870 PMC3486905

[b27] Bahia M. T. . Antitrypanosomal activity of fexinidazole metabolites, potential new drug candidates for Chagas disease. Antimicrob. Agents Chemother. 58, 4362–4370 (2014). 24841257 10.1128/AAC.02754-13 PMC4136024

[b28] Lewis M. D. . Recent, independent and anthropogenic origins of *Trypanosoma cruzi* hybrids. PLoS Negl. Trop. Dis. 5, e1363 (2011). 22022633 10.1371/journal.pntd.0001363 PMC3191134

[b29] Lewis M. D., Fortes Francisco A., Taylor M. C., Jayawardhana S. & Kelly J. M. Host and parasite genetics shape a link betweens*Trypanosoma cruzi* infection dynamics and chronic cardiomyopashs. *Cell. Microbiol.* 18, 1429–1443 (2016). 10.1111/cmi.12584 PMC5031194 26918803

[b30] Torreele E. . Fexinidazole – a new oral nitroimidazole drug candidate entering clinical development for the treatment of sleeping sickness. PLoS Negl. Trop. Dis. 4, e923 (2010). 21200426 10.1371/journal.pntd.0000923 PMC3006138

[b31] Chatelain E. & Konar N. Translational challenges of animal models in Chagas disease drug development: a review. J. Drug Des. Devel. Ther. 9, 4807–4823 (2015). 10.2147/DDDT.S90208 PMC4548737 26316715

[b32] Buckner F. Experimental chemotherapy and approaches to drug discovery for *Trypanosoma cruzi* infection. Adv. Parasitol. 75, 89–119 (2011). 21820553 10.1016/B978-0-12-385863-4.00005-8

[b33] Canavaci A. M. . *In vitro* and *in vivo* high-throughput assays for the testing of anti-*Trypanosoma cruzi* compounds. PLoS Negl. Trop. Dis. 4, e740 (2010). 20644616 10.1371/journal.pntd.0000740 PMC2903469

[b34] Romanha A. J. . *In vitro* and *in vivo* experimental models for drug screening and development for Chagas disease. Mem. Inst. Oswaldo Cruz 105, 233–238 (2010). 20428688 10.1590/s0074-02762010000200022

[b35] http://www.dndi.org/diseases-projects/chagas/chagas-target-product-profile/.

[b36] Assíria Fontes Martins T. . Benznidazole/itraconazole combination treatment enhances anti-*Trypanosoma cruzi* activity in experimental Chagas disease. PLoS One 10, e0128707 (2015). 26076455 10.1371/journal.pone.0128707 PMC4468053

[b37] Gruendling A. P. . Impact of benznidazole on infection course in mice experimentally infected with *Trypanosoma cruzi* I, II, and IV. Am. J. Trop. Med. Hyg. 92, 1178–1189 (2015). 25940197 10.4269/ajtmh.13-0690 PMC4458823

[b38] Molina-Berríos A. . Benznidazole prevents endothelial damage in an experimental model of Chagas disease. Acta Trop. 127, 6–13 (2013). 23529066 10.1016/j.actatropica.2013.03.006

[b39] Davies C. . Basombrío MA. Hydroxymethylnitrofurazone is active in a murine model of Chagas’ disease. Antimicrob. Agents Chemother. 54, 3584–3589 (2010). 20566772 10.1128/AAC.01451-09 PMC2934987

[b40] Villar J. C. . Trypanocidal drugs for chronic asymptomatic *Trypanosoma cruzi* infection. Cochrane Database Syst. Rev. 5, CD003463 (2014). 24867876 10.1002/14651858.CD003463.pub2 PMC7154579

[b41] Hamers R. L., van Gool T. & Goorhuis A. Benznidazole for chronic Chagas’ cardiomyopathy. N. Engl. J. Med. 374, 188 (2016). 10.1056/NEJMc1514453 26760093

[b42] Cordeiro M. A. Benznidazole for chronic Chagas’ cardiomyopathy. N. Engl. J. Med. 374, 188–189 (2016). 10.1056/NEJMc1514453 26760094

[b43] Urbina J. A., Gascon J. & Ribeiro I. Benznidazole for chronic Chagas’ cardiomyopathy. N. Engl. J. Med. 374, 189 (2016). 10.1056/NEJMc1514453 26760095

[b44] Morillo C. A., Marin-Neto J. A. & Avezum A. Benznidazole for chronic Chagas’ cardiomyopathy. N. Engl. J. Med. 374, 189–190 (2016). 10.1056/NEJMc1514453 26760092

[b45] http://www.dndi.org/diseases-projects/portfolio/fexinidazole-chagas/.

[b46] Hall B. S. & Wilkinson S. R. Activation of benznidazole by trypanosomal type I nitroreductases results in glyoxal formation. Antimicrob. Agents Chemother. 56, 115–123 (2012). 22037852 10.1128/AAC.05135-11 PMC3256028

[b47] Hall B. S., Bot C. & Wilkinson S. R. Nifurtimox activation by trypanosomal type I nitroreductases generates cytotoxic nitrile metabolites. J. Biol. Chem. 286, 13088–13095 (2011). 21345801 10.1074/jbc.M111.230847 PMC3075655

[b48] Isola E. L., Lammel E. M. & Gonzalez Cappa S. M. *Trypanosoma cruzi*: differentiation after interaction of epimastigotes and *Triatoma infestans* intestinal homogenate. Exp. Parasitol. 62, 329–335 (1986). 3023131 10.1016/0014-4894(86)90039-1

[b49] Coteron J. M. . Structure-guided lead optimization of triazolopyrimidine-ring substituents identifies potent *Plasmodium falciparum* dihydroorotate dehydrogenase inhibitors with clinical candidate potential. J. Med. Chem. 54, 5540–6551 (2011). 21696174 10.1021/jm200592f PMC3156099

